# Ethyl pyruvate attenuates acetaminophen-induced liver injury and prevents cellular injury induced by *N*-acetyl-*p*-benzoquinone imine

**DOI:** 10.1016/j.heliyon.2018.e00521

**Published:** 2018-02-01

**Authors:** Minako Nagatome, Yuki Kondo, Daisuke Kadowaki, Yusuke Saishyo, Mitsuru Irikura, Tetsumi Irie, Yoichi Ishitsuka

**Affiliations:** a Department of Clinical Chemistry and Informatics, Graduate School of Pharmaceutical Sciences, Kumamoto University, 5-1 Oe-honmachi, Chuo-ku, Kumamoto 862-0973, Japan; b Laboratory of Clinical Pharmaceutics, Faculty of Pharmaceutical Science, Sojo University, 4-22-1 Ikeda, Kumamoto 860-0082, Japan; c Laboratory of Evidence-Based Pharmacotherapy, College of Pharmaceutical Sciences, Daiichi University, 22-1 Tamagawa-Cho, Minami-Ku, Fukuoka 815-8511, Japan; d Center for Clinical Pharmaceutical Sciences, Faculty of Pharmaceutical Sciences, Kumamoto University, 5-1 Oe-honmachi, Chuo-ku, Kumamoto 862-0973, Japan

**Keywords:** Pathology, Toxicology

## Abstract

Acetaminophen, a common analgesic/antipyretic, is a frequent cause of acute liver failure in Western countries. The development of an effective cure against acetaminophen hepatotoxicity is crucial. Ethyl pyruvate, an ethyl ester derivative of pyruvic acid, has been identified as a possible candidate against acetaminophen hepatotoxicity in animal experiments. However, the mode of the hepatoprotective action of ethyl pyruvate remains unclear. We examined the hepatoprotective effect of ethyl pyruvate against hepatocyte injury and oxidative stress in a mouse model of acetaminophen hepatotoxicity. In addition, to examine whether ethyl pyruvate has direct hepatocellular protection against acetaminophen hepatotoxicity to counteract the influence of inflammatory cells, such as macrophages, we examined the effects of ethyl pyruvate on cellular injury induced by *N*-acetyl-*p*-benzoquinone imine, a toxic metabolite of acetaminophen, in a human hepatocyte cell line, HepG2 cells. Treatment with ethyl pyruvate significantly prevented increases in serum transaminase levels and hepatic centrilobular necrosis induced with an acetaminophen overdose in mice in a dose-dependent manner. Although hepatic DNA fragmentation induced by acetaminophen was also attenuated with ethyl pyruvate, nitrotyrosine formation was not inhibited. Ehyl pyruvate significantly attenuated mitochondria dehydrogenase inactivity induced by *N*-acetyl-*p*-benzoquinone imine in HepG2 cells. The attenuating effect was also observed in a rat hepatocyte cell line. Increases in annexin V and propidium iodide-stained cells induced by *N*-acetyl-*p*-benzoquinone imine were prevented with ethyl pyruvate in HepG2 cells. Pyruvic acid, a parent compound of ethyl pyruvate, tended to attenuate these changes. The results indicate that ethyl pyruvate has direct hepatocellular protection against *N*-acetyl-*p*-benzoquinone imine induced injury observed in acetaminophen overdose. The *in vivo* and *in vitro* results suggest that ethyl pyruvate attenuates acetaminophen-induced liver injury via, at least in part, its cellular protective potential.

## 1 Introduction

Acetaminophen (*N*-acetyl-*p*-aminophenol, paracetamol, APAP) is a common analgesic/antipyretic in numerous medicinal and over-the-counter drug formulations. Although APAP shows few side effects at therapeutic doses, overdoses can produce severe hepatic injury. APAP-induced liver injury is the most frequent cause of acute liver failure in the United States [1, 2], the United Kingdom [3, 4] and other countries [5, 6]. APAP hepatotoxicity is characterized by extensive centrilobular necrosis and infiltration of inflammatory cells [7, 8]. The hepatocellular necrosis and inflammation is initiated by a reactive metabolite of APAP, *N*-acetyl-*p*-benzoquinone imine (NAPQI), mainly produced by cytochrome P450 (CYP) 2E1 [9, 10]. NAPQI seems to injure hepatocytes with glutathione depletion, oxidative and nitrosative stress, and inflammation. To detoxify NAPQI, the glutathione precursor *N*-acetylcysteine (NAC) was identified and has been used as the only approved antidote against APAP hepatotoxicity. However, NAC sometimes shows limited hepatoprotective effects in APAP-overdose patients because of delay in starting treatment. Therefore, the development of a novel effective antidote is needed.

Numerous researchers have looked for effective candidate compounds against APAP-induced liver injury [11, 12, 13, 14, 15, 16, 17]. Ethyl pyruvate (EtPy), an ethyl ester derivative of pyruvic acid, was identified as a potentially good compound by Yang et al. [18]. EtPy has been developed to be a highly membrane-permeable derivative of pyruvic acid, a glycolysis intermediate with multiple physiological properties including potent anti-inflammatory and anti-oxidative effects [19, 20, 21]. Yang et al. reported that EtPy inhibited increases in transaminase levels and tumor necrosis factor (TNF)-α concentrations in serum, hepatic centrilobular necrosis, and infiltration of inflammatory cells in a mouse model of APAP-overdose, and suggested the preventive effects of EtPy against APAP hepatitis in mice. Suppressive effects of EtPy on activated inflammatory cells, such as macrophages and neutrophils, and reduction of cytokine release from cells, such as TNF-α, may be involved in the hepatoprotective mechanism of EtPy. However, EtPy also has a scavenger action against reactive oxygen species, such as hydrogen peroxide, and cytoprotective potential against cellular injury through anti-apoptotic and necroptotic effects [22, 23, 24]. Therefore, it is suggested that the antioxidative action and direct hepatocyte protection by EtPy against NAPQI-induced hepatocyte injury without inhibiting inflammatory cells should be considered. Little is reported on the hepatoprotective effects of EtPy in APAP-induced liver injury models.

This study was conducted to evaluate the hepatoprotective action of EtPy against APAP hepatotoxicity with regard to its antioxidative and cytoprotective effects. Using a mouse model of APAP hepatotoxicity, we examined the effects of EtPy on the increase in serum transaminase levels, centrilobular necrosis, DNA fragmentation, and nitrotyrosine formation in the liver. We also conducted an *in vitro* study to determine the scavenger potential of EtPy against reactive oxygen species, such as hydroxyl radicals and peroxynitrite, which play important roles in the development of APAP liver injury. In addition, we evaluated whether EtPy shows direct hepatocyte protection during APAP-induced liver injury using an *in vitro* system to counteract the influences of inflammatory cells, such as macrophages and neutrophils. We examined the effects of EtPy on NAPQI-induced cell injury in a representative hepatocellular model mainly using mainly HepG2 cells, a human hepatoma cell line. Then we compared the effects of EtPy with pyruvic acid (PyA), a parent compound of EtPy, and phosphopyruvic acid (PEP), a precursor of PyA in glycolysis, against NAPQI-induced cell injury.

## 2 Materials and methods

### 2.1 Reagents

EtPy was purchased from Alfa Aesar (Ward Hill, MA) and Sigma-Aldrich (St. Louis, MO). Sodium pyruvate (PyA) was obtained from Nacalai Tesque (Kyoto, Japan). Sodium phosphopyruvate was kindly donated by Ube Kousan (Yamaguchi, Japan). APAP, *N*-acetyl cyctein (NAC) and NAPQI were purchased from Sigma-Aldrich. A cell counting kit was obtained from Dojindo Laboratories (Kumamoto, Japan). An annexin V-FITC apoptosis detection kit was purchased from R&D Systems (Minneapolis, MN). All other reagents and solvents were of reagent grade. Deionized and distilled bio-pure grade water was used in the study.

### 2.2 Animal experiments

#### 2.2.1 Animals

Male wild-type C57BL/6JJcl mice (8–10 weeks old) (CLEA Japan, Shiga, Japan) were used. Animals were housed in cages under controlled conditions at 24 °C on a 12-h light/dark cycle and had free access to food and water. All experimental procedures conformed with the Animal Use Guidelines of the Committee for Ethics on Animal Experiments at Kumamoto University. The study protocol was approved by the ethical committee (the name of committee: “Animal Care and Use Committee of Kumamoto University”, The approval No.: B27-131 and A29-132).

#### 2.2.2 Experimental settings and treatments

**Fig. 1:**
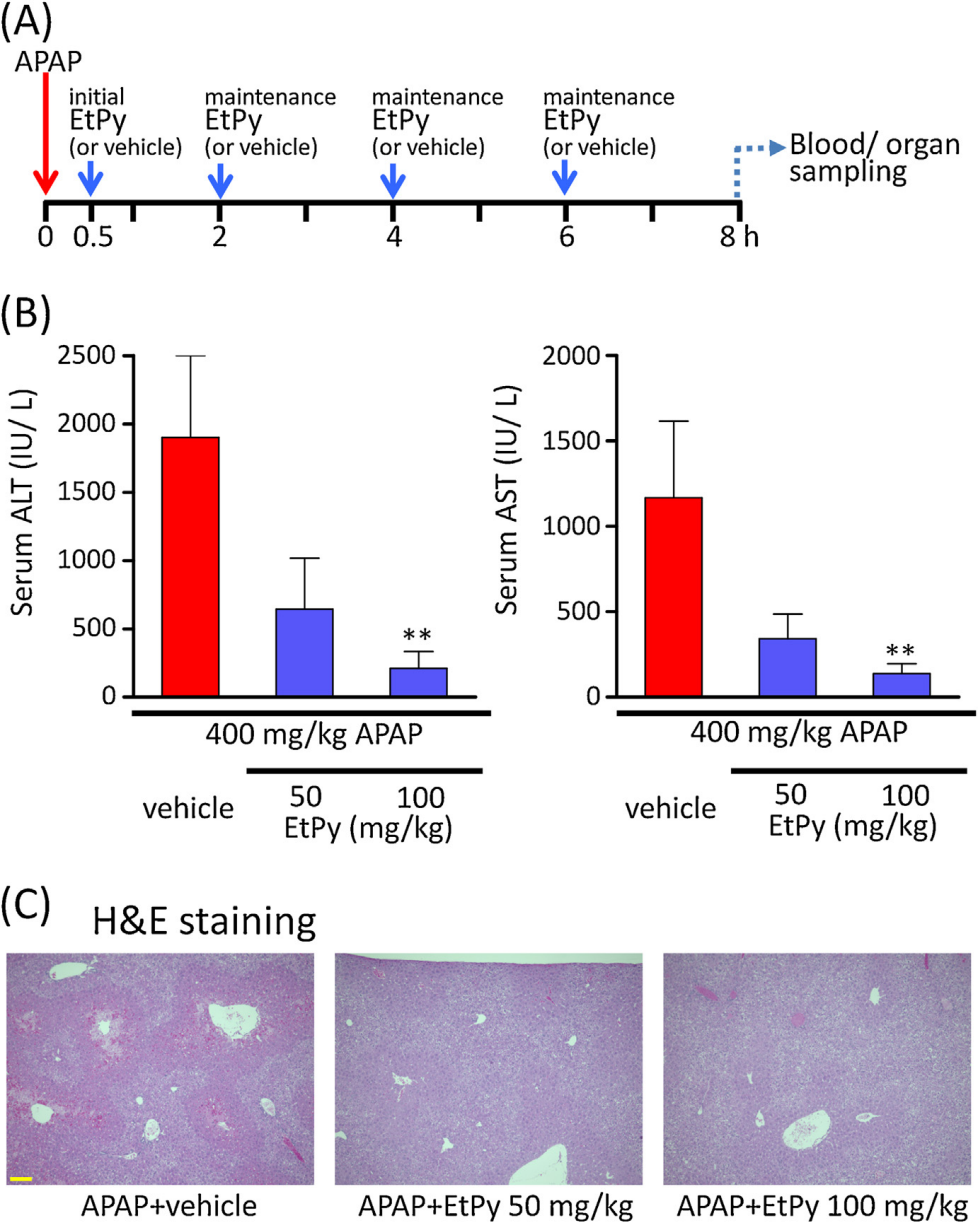
Attenuating effects of ethyl pyruvate (EtPy) against acetaminophen (APAP)-induced liver injury in mice. (A) Treatment schedule of APAP and EtPy. An intraperitoneal injection of APAP (400 mg/kg) was administered at 0 h. EtPy was dissolved in Ringer’s solution and administered at 0.5, 2, 4, and 6 h after the APAP injection (four times in total). In the EtPy 50 mg/kg group, mice were treated with 20 mg/kg EtPy at 0.5 h after the APAP injection as the initial dose. Then at 2, 4, and 6 h after APAP injection, 10 mg/kg of EtPy were administered. In the 100 mg/kg group, an initial dose of 40 mg/kg EtPy was administered and then maintained at 20 mg/kg. (B) Effects of EtPy on the increase in serum alanine aminotransferase (ALT) and aspartate transaminase (AST) levels in APAP overdosed mice. A significant decrease was observed in the EtPy 100 mg/kg group compared with the vehicle group. Each bar represents mean ± SEM, n = 6. ** *p* < 0.01 compared with the vehicle-treated group. (C) Representative histological images of APAP overdosed mice. Significant centrilobular necrosis with bleeding was observed in the vehicle-treated group. Although slight hepatocellular swelling was present, few necrotic hepatocytes were observed in the groups treated with EtPy. Scale bar: 100 μm.

The mouse model of APAP hepatitis was developed as described previously [13, 25, 26]. Briefly, APAP was dissolved in phosphate-buffered saline at 55–60 °C and prepared in a 2% solution. An overdose of APAP (400 mg/kg) was intraperitoneally injected into mice to induce hepatotoxicity. EtPy was dissolved in Ringer’s solution and administered intraperitoneally at 0.5, 2, 4, and 6 h after the APAP injection. Animals were divided by three groups: (1) a vehicle group; (2) a low-dose EtPy (50 mg/kg) group; and (3) a high-dose EtPy (100 mg/kg) group. A treatment schedule is provided in Fig. 1A. Mice in the low-dose EtPy (50 mg/kg) group were treated with 20 mg/kg EtPy at 0.5 h after the APAP injection, and 10 mg/kg of EtPy (three times in total) at 2, 4, and 6 h after the injection. Mice in the high-dose EtPy (100 mg/kg) group were treated with 40 mg/kg EtPy at 0.5 h after the APAP injection, and 20 mg/kg of EtPy (three times in total) at 2, 4, and 6 h after the injection. Mice in the vehicle group were administered Ringer’s solution (1 μL/g body weight) at 0.5, 2, 4, and 6 h after the APAP injection. Mice were killed at 8 h after the APAP injection and blood and tissue samples were collected.

#### 2.2.3 Evaluation of liver injury

The protective effects of EtPy were evaluated by examining serum alanine aminotransferase (ALT) and aspartate transaminase (AST) levels, and histological analysis using hematoxylin and eosin staining of samples. DNA fragmentation and nitrotyrosine formation were evaluated using the terminal deoxynucleotidyl transferase dUTP nick end labeling (TUNEL) assay and immunohistochemistry, respectively. The assay was performed as described previously [13, 26]. Blood samples was centrifuged at 4000 *× g* at 4 °C for 10 min after coagulation and serum was collected. Serum ALT and AST levels were determined using a bio-analyzer (SPOTCHEM EZ SP-4430; ARKRAY, Kyoto, Japan). Liver tissue samples were fixed in 10% neutral buffered formalin and embedded in paraffin. Microtome sections (3-μm thick) were prepared and stained with hematoxylin and eosin. The TUNEL assay was performed using the ApopTag^®^ Peroxidase In Situ Apoptosis Detection Kit (Merck Millipore, Billerica, MA) according to the manufacturer’s instructions. To evaluate nitrotyrosine formation, immunohistochemical analysis using an anti-nitrotyrosine polyclonal antibody (Merck Millipore) was performed. Microtome sections were incubated overnight at 4 °C with the anti-nitrotyrosine antibody (1:500 dilution) and stained with Histofine^®^ Simple Stain MAX PO (Nichirei Biosciences, Tokyo, Japan). Following the wash step, 3,3-diaminobenzidine was applied to sections and sections were incubated with Mayer’s hematoxylin. Histological evaluation was performed in an unblinded manner.

### 2.3 Cell experiments

#### 2.3.1 Cell line and cell culture

HepG2 cells (No. RCB1648), a human hepatoma cell line, was purchased from RIKEN BioResource Center (Ibaraki, Japan). HepG2 cells were cultured in minimum essential medium (MEM) containing 10% fetal bovine serum (FBS), 100 IU/mL penicillin, 100 mg/mL streptomycin, and 0.1 mM non-essential amino acids (Thermo Fisher Scientific, Waltham, MA). Cells were cultured under 5% CO_2_ and 95% air at 37 °C. RLC-16 cells (No. RCB1474), a rat hepatocyte cell line, was purchased from RIKEN BioResource Center (Ibaraki, Japan) and cultured in same conditions as HepG2 cells.

#### 2.3.2 Development of the model of NAPQI-induced hepatocellular injury

Cellular injury induced by NAPQI was evaluated using methods described previously [12, 27]. HepG2 or RLC cells were seeded at 1 × 10^4^ cells/well into a 96-well plate. After 24 h to allow cells to adhere, medium was replaced with fresh medium containing NAPQI, with or without test compounds, such as EtPy, PyA, or PEP. NAC was used as the positive control against NAPQI-induced cellular injury. Mitochondrial dehydrogenase activity was estimated 24 or 48 h after NAPQI treatment using a modified MTT assay (the water-soluble tetrazolium salt (WST-1) assay) and a cell counting kit (Dojindo Laboratories). Apoptotic or necrotic-like cellular injuries were measured using an annexin V-FITC apoptosis detection kit. HepG2 cells were placed at 1 × 10^5^ cells/dish into a 35-mm^2^ dish for 24 h. Culture medium was replaced with fresh medium containing NAPQI with or without the test compounds. Cells were stained using an annexin V-FITC apoptosis detection kit and analyzed using a BD FACSCalibur™ (Becton, Dickinson and Company, Franklin Lakes, NJ).

### 2.4 Evaluation of peroxynitrite and hydroxyl radicals scavenging activity

To determine the antioxidative effects of EtPy, we evaluated the scavenging activity against peroxynitrite and hydroxyl radicals (•OH), which play important roles in the development of APAP hepatotoxicity. The methods have been described previously [28]. We used dihydrorhodamine, a reduced form of rhodamine, as a probe for peroxynitrite activity to examine the scavenging effect of EtPy. Peroxynitrite was diluted with 0.3 M NaOH to give a 7-mL solution. A mixture containing 100 mL sodium phosphate buffer (pH 7.4), 0.3 mg/mL gelatin, 25 mL dihydrorhodamine, and EtPy was pre-incubated at 37 °C for 5 min, and the reaction was started by adding 200 mL of the mixture to 2 mL of peroxynitrite solution. After incubation at 37 °C for 5 min, the generated rhodamine was estimated using the above-described fluorescence measurements. A peroxynitrite-dependent increase in fluorescence was then converted into the rhodamine concentration from the external rhodamine standard.

•OH was generated using an H_2_O_2_/ultraviolet (UV) radiation system and was measured by electron paramagnetic resonance (EPR) spin-trapping with 5,5-Dimethyl-1-pyrroline N-oxide (DMPO; Enzo Life Sciences, Farmingdale, NY). The reaction mixture contained 100 μM diethylenetriamine-*N,N,N′,N″,N″*-pentaacetic acid, 9 mM DMPO, and 500 μM H_2_O_2_ in the absence or presence of EtPy. This mixture was immediately transferred to the EPR flat cells and irradiated with UV (254 nm) for 30 s. EPR spectra were obtained immediately after UV-irradiation and were recorded at room temperature on a JES-TE 200 EPR spectrometer (JEOL, Tokyo, Japan). After recording EPR spectra, the signal intensities of the DMPO—OH adducts were normalized to that of a manganese oxide (Mn^2+^) signal, in which Mn^2+^ served as an internal control. An EPR spectrum of DMPO spin adducts of •OH was generated, and the relative quantification of the scavenging activity was evaluated using DMPO spin adduct intensities of •OH.

### 2.5 Statistical analysis

Statistical analyses were performed using GraphPad Prism (ver. 5.01; GraphPad Software, San Diego, CA). Multiple comparisons were used to examine the statistical significance of the data. When uniform variance was identified using Bartlett’s analysis (*P* > 0.05), one-way analysis of variance (ANOVA) was used to test for statistical significance. When significant differences (*P* < 0.05) were identified, data were further analyzed using the Tukey (or Tukey–Kramer) multiple range test, as appropriate.

## 3 Results

### 3.1 Attenuating effects of EtPy against APAP-induced liver injury in mice

To confirm the effects of EtPy against APAP hepatotoxicity, we used a mouse model of liver injury induced by an overdose of APAP. Mice were treated with multiple doses of EtPy after the APAP injection, for totals of 50 and 100 mg/kg of EtPy for each group (Fig. 1A). Treatment with EtPy significantly reduced serum ALT and AST levels at 8 h after the APAP injection (Fig. 1B). In the vehicle treatment group, numerous areas of centrilobular necrosis with bleeding were observed, which is a typical manifestation of APAP hepatotoxicity (Fig. 1C; left). Although slight histological changes, such as swelling of hepatocytes around central veins, were observed, little centrilobular necrosis was visible in both EtPy groups (Fig. 1C).

**Fig. 2:**
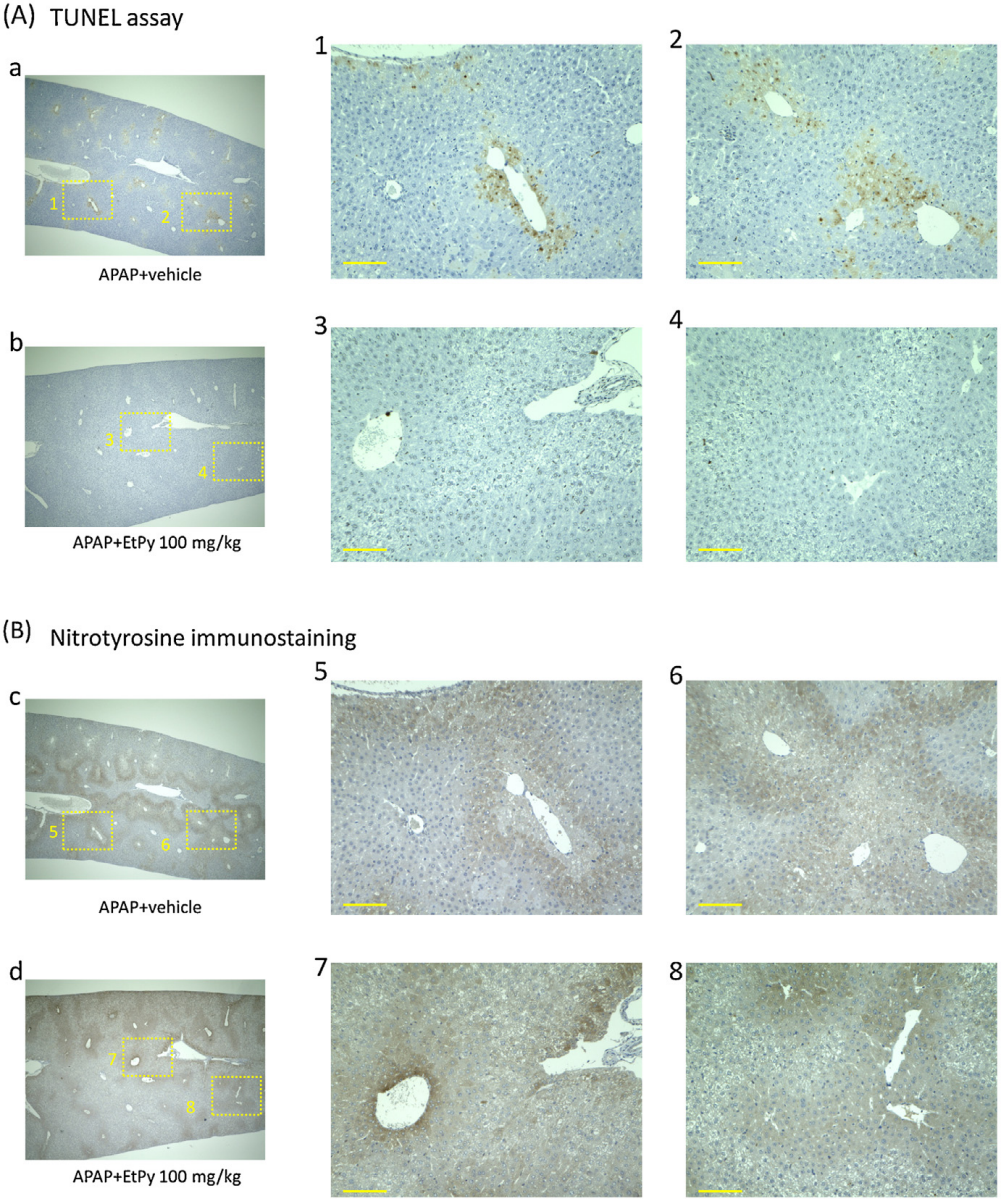
Effects of ethyl pyruvate (EtPy) on DNA fragmentation and nitrotyrosine adduct formation in mouse livers treated with an overdose of acetaminophen (APAP). Representative images of (A) TUNEL staining and (B) immunohistochemistry staining for nitrotyrosine in hepatic histological sections. Liver samples were collected at 8 h after APAP injection (see Fig. 1A). Panels a–d are low-magnification images for each group. Panel 1–8 are high-magnification images for the squares marked in a–d. TUNEL-stained cells were identified in many lobes in the APAP + vehicle group (Panel a, 1 and 2). EtPy treatment inhibited changes (Panel b, 3 and 4). As shown in Panel c, 5 and 6, extensive anti-nitrotyrosine-positive cells were observed in the APAP + vehicle group. Contrary to the results of the TUNEL assay, a significant decrease was not observed in the APAP + EtPy 100 mg/kg group. Scale bar: 100 μm.

To investigate the effects of EtPy on nuclear DNA fragmentation, the TUNEL assay was performed. As shown in Fig. 2A, many lobes in the vehicle group showed some TUNEL-stained cells. In contrast, only a few TUNEL-positive cells were visible in the EtPy groups.

Hepatic nitrotyrosine adduct formation was evaluated using immunohistochemical staining for nitrotyrosine in hepatic histological sections. Extensive nitrotyrosine staining of centrilobular hepatocytes was observed in all groups (Fig. 2B). Treatment with EtPy did not seem to inhibit the nitrotyrosine adduct formation induced by APAP.

### 3.2 Preventive effects of EtPy on the decrease in mitochondrial dehydrogenase activity induced by NAPQI in HepG2 and RLC cells

**Fig. 3:**
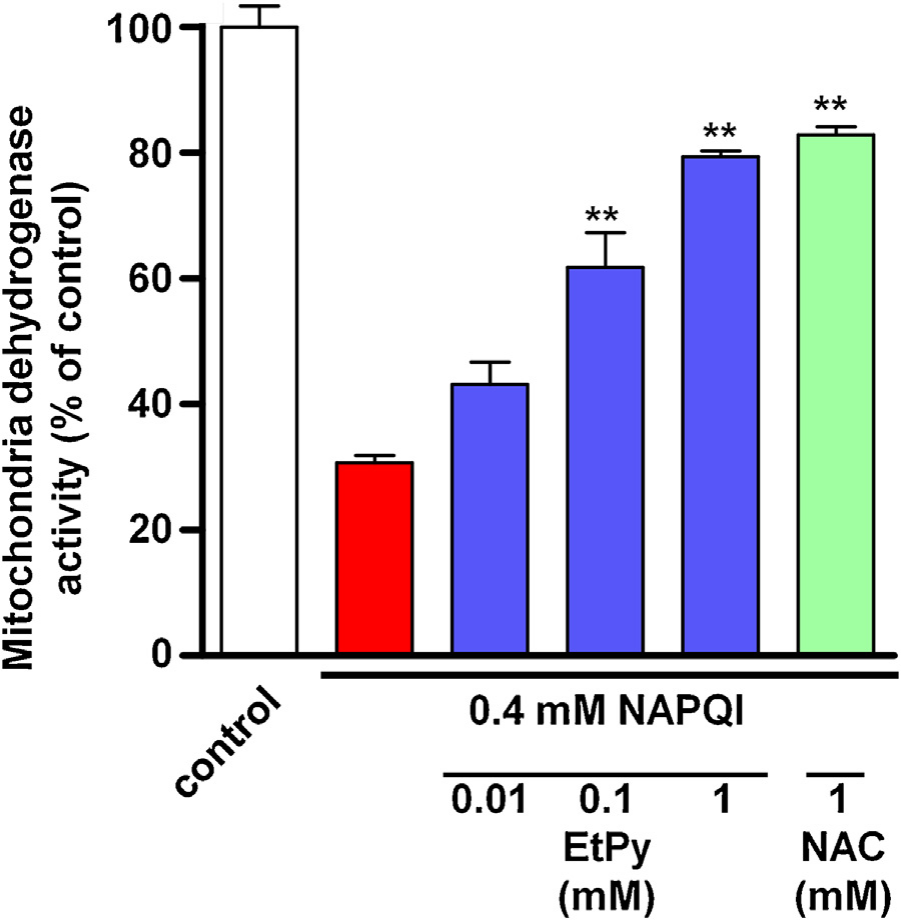
Dose-dependent effects of ethyl pyruvate (EtPy) on the decrease in mitochondrial dehydrogenase activity induced by *N*-acetyl-*p*-benzoquinone imine (NAPQI) in HepG2 cells. HepG2 cells were treated with 0.4 mM NAPQI. Mitochondrial dehydrogenase activity was measured in cells either in the presence or absence of EtPy (0.01–1 mM). A significant decrease in mitochondrial dehydrogenase activity was induced in the NAPQI-treated group (red bar) compared with the untreated control group (white bar). EtPy (blue bars) showed preventive effects against the decrease in mitochondrial dehydrogenase activity in a dose-dependent manner. Mitochondrial dehydrogenase activity was measured using the water-soluble tetrazolium salt (WST-1) assay. Data are expressed as mean ± SEM, n = 6. **p < 0.01 compared with the NAPQI-treated group.

**Fig. 4:**
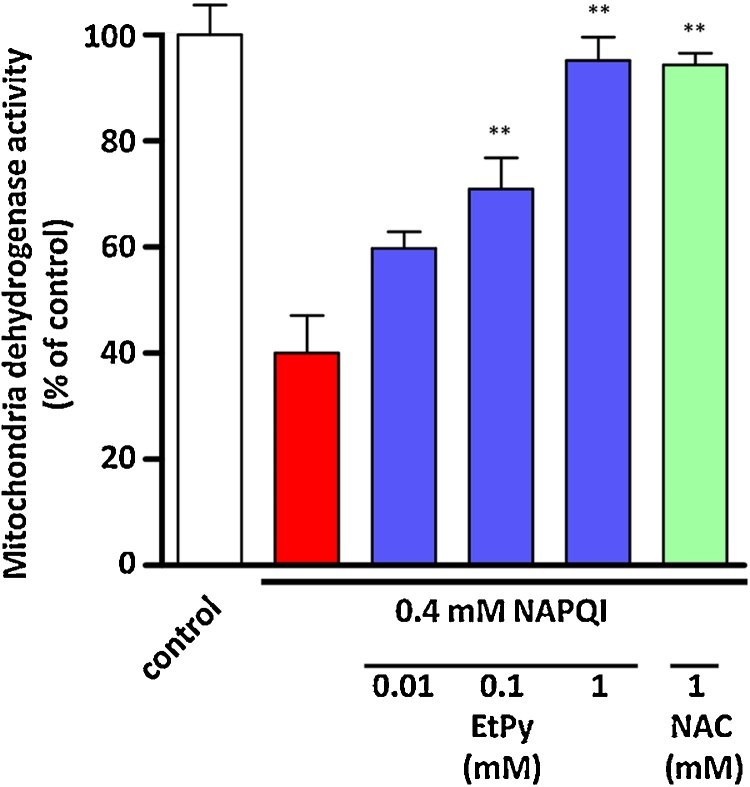
Effects of ethyl pyruvate (EtPy) on the decrease in mitochondrial dehydrogenase activity induced by *N*-acetyl-*p*-benzoquinone imine (NAPQI) in a rat hepatocyte cell line, RLC cells. RLC cells were treated with 0.25 mM NAPQI. Mitochondrial dehydrogenase activity was measured in cells either in the presence or absence of EtPy (0.01–1 mM). A significant decrease in mitochondrial dehydrogenase activity was induced in the NAPQI-treated group (red bar) compared with the untreated control group (white bar). EtPy (blue bars) showed preventive effects against the decrease in mitochondrial dehydrogenase activity in a dose-dependent manner. Mitochondrial dehydrogenase activity was measured using the water-soluble tetrazolium salt (WST-1) assay. Data are expressed as mean ± SEM, n = 4. ** *p* < 0.01 compared with the NAPQI-treated group.

To evaluate the cytoprotective potential of EtPy against hepatocellular injury induced by NAPQI, a toxic metabolite of APAP, we examined the effects of EtPy on mitochondrial dehydrogenase activity in NAPQI-treated HepG2 and RLC cells. A significant decrease in mitochondrial dehydrogenase activity was observed with NAPQI treatment in HepG2 cells (Fig. 3A). Treatment with EtPy significantly prevented the decrease in mitochondrial dehydrogenase activity induced by NAPQI in a dose-dependent manner in the cells. These preventive effects exerted by 1 mM EtPy were comparable with the effects of 1 mM NAC, a therapeutic antidote against APAP hepatotoxicity. The same preventive effect of EtPy was observed in another hepatocellular model using a rat hepatocellular cell line (RLC cells) (Fig. 4).

### 3.3 Comparison of the preventive effects of pyruvate derivatives on the decrease in mitochondrial dehydrogenase activity induced by NAPQI in HepG2 cells

**Fig. 5:**
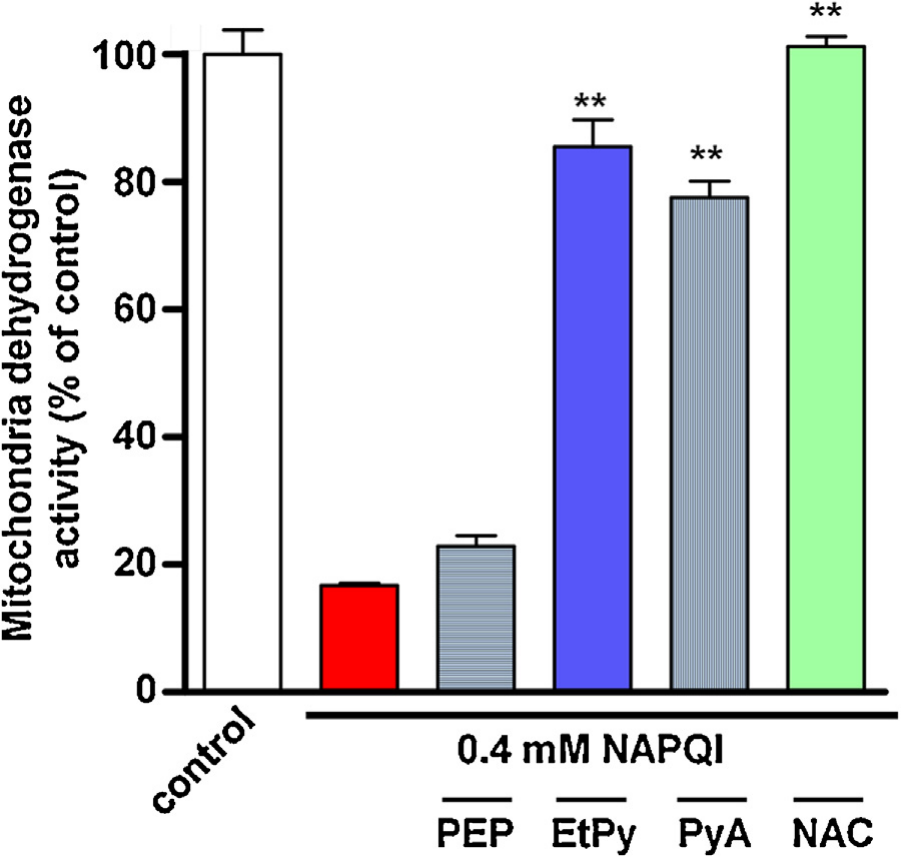
Comparative effects of pyruvic acid (PyA) derivatives on the decrease in mitochondrial dehydrogenase activity induced by *N*-acetyl-*p*-benzoquinone imine (NAPQI) in HepG2 cells. HepG2 cells were treated with 0.4 mM NAPQI either in the presence or absence of PyA derivatives (1 mM), such as phosphopyruvic acid (PEP) (grey), ethyl pyruvate (EtPy) (blue), and PyA (grey). Mitochondrial dehydrogenase activity was measured using the water-soluble tetrazolium salt (WST-1) assay. Data are expressed as mean ± SEM, n = 6. **p < 0.01 compared with the NAPQI-treated group.

We compared the cytoprotective effects of EtPy with the parent compound, PyA, and a glycolysis intermediate, PEP, a precursor of PyA, in NAPQI-treated HepG2 cells. The 1 mM PyA group showed a significant inhibition in the decrease of mitochondrial dehydrogenase activity induced by NAPQI in HepG2 cells, as well as the EtPy and NAC groups (Fig. 5). However, 1 mM of PEP did not exert any effect on NAPQI-induced mitochondrial dehydrogenase inactivity in HepG2 cells.

### 3.4 Annexin V and propidium iodide analysis in NAPQI-treated HepG2 cells

**Fig. 6:**
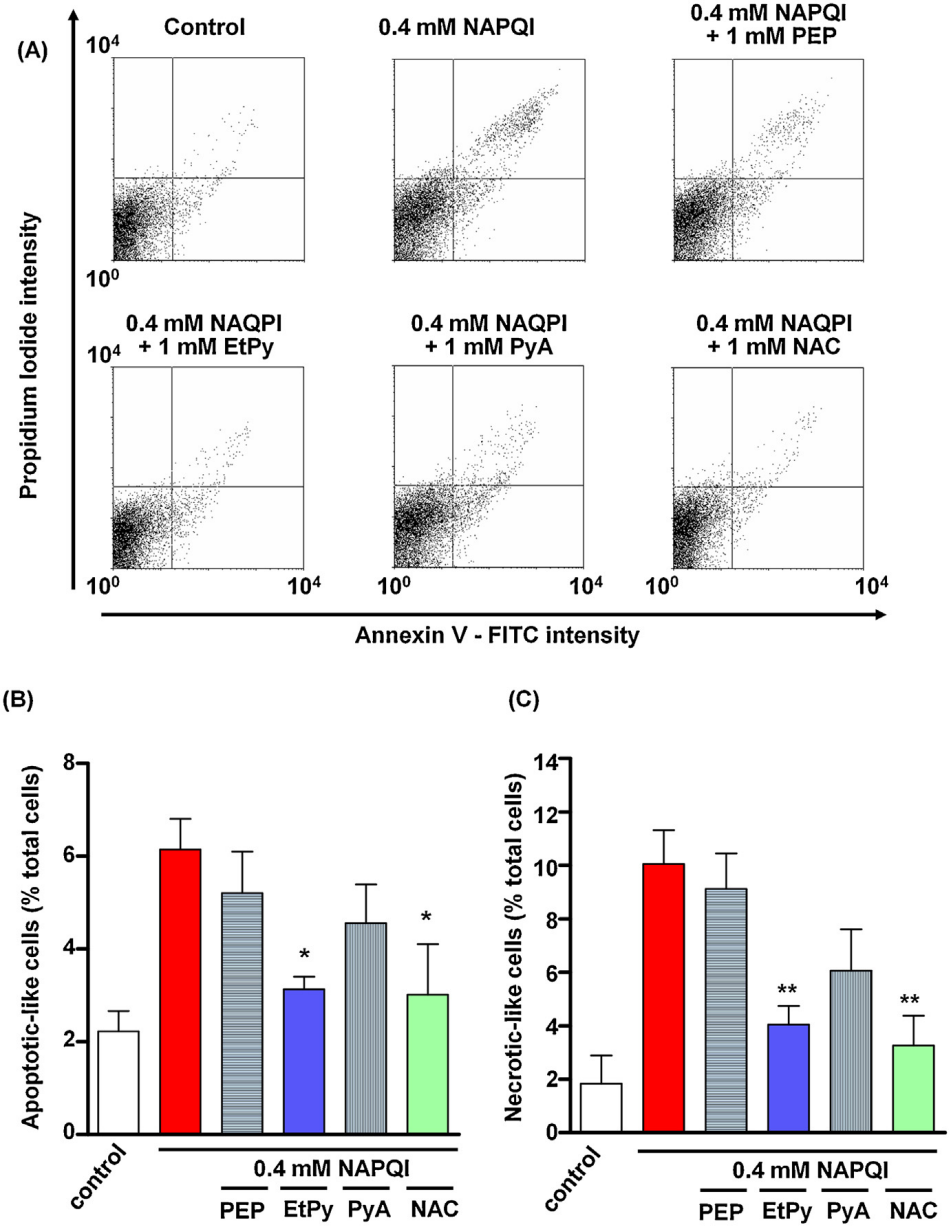
Effects of pyruvic acid (PyA) derivatives on apoptotic and necrotic-like cell death induced by *N*-acetyl-*p*-benzoquinone imine (NAPQI) in HepG2 cells. NAPQI (0.4 mM)-exposed HepG2 cells were treated with or without 1 mM PyA derivatives (phosphopyruvic acid (PEP), ethyl pyruvate (EtPy), and PyA) for 24 h. Cells were then stained with annexin V-FITC/propidium iodide assay, and analyzed by flow cytometry. (A) Data were plotted as annexin V-FITC intensity (X axis) *vs.* propidium iodide intensity (Y axis). Annexin+/propidium iodide− (down right) and annexin+/PI+ (up right) are apoptotic-like cells and necrotic-like cells, respectively. Quantitative analysis of (B) apoptotic-like and (C) necrotic-like cells are shown. The number of cells is expressed as a percentage of the total cell number. Data are expressed as mean ± SEM, n = 3. *p < 0.05, **p < 0.01 compared with the NAPQI-treated group.

We evaluated the effects of pyruvate derivatives on cellular apoptosis- or necrotic-like cell death induced by NAPQI in HepG2 cells. Representative data from FACS analysis of annexin V-FITC- and propidium iodide-stained HepG2 cells are shown in Fig. 6A. Significant increases in both annexin V and propidium iodide fluorescence intensities were observed with NAPQI treatment (Fig. 6A, upper middle) compared with the control group (Fig. 6A, upper left). Treatment with EtPy (1 mM) attenuated the increase in annexin V and propidium iodide fluorescence intensities induced by NAPQI (Fig. 6A, lower left), with statistical significances being observed in quantitative measurements of numbers of annexin V-stained cells (Fig. 6B) and annexin V–propidium iodide double-stained cells (Fig. 6C). The attenuating effects were comparable with the effects of NAC (Fig. 6A, lower right). Although PyA (1 mM) also tended to prevent the increase in annexin V and/or propidium iodide fluorescence, no statistically significant differences were observed. PEP did not show any significant effects on annexin V and propidium iodide fluorescence intensities in NAPQI-treated HepG2 cells.

### 3.5 Evaluation of peroxynitrite and hydroxyl radicals scavenging activity

**Fig. 7:**
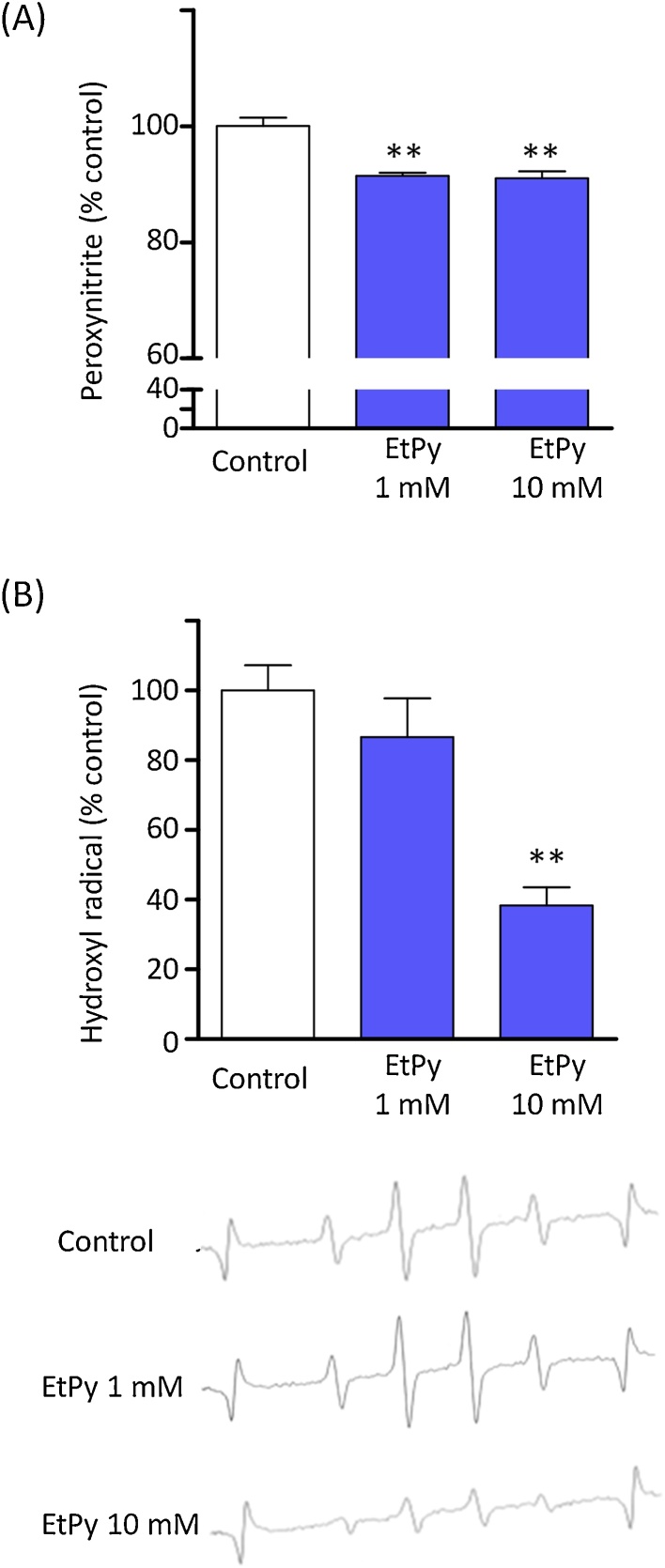
Evaluation of scavenging activity of ethyl pyruvate (EtPy) against (A) peroxynitrite and (B) hydroxyl radicals. (A) Scavenging activity of EtPy against peroxynitrite. The peroxynitrite-dependent increase in fluorescence was converted into a rhodamine concentration from the external rhodamine standard. The fluorescence intensity of rhodamine is expressed as relative (%) to vehicle control. One and 10 mM of EtPy showed scavenged peroxynitrite. The effects were statistically significant, but the reducing levels by just approximately 10%. Each bar represents mean ± SEM, *n* = 6. ** *p ≤* 0.01, compared with the 0 mM group. (B) Scavenging activity of EtPy on hydroxyl radicals was assayed using the DMPO–OH EPR signal. Left bar graph shows the scavenging activity of EtPy expressed by the decrease in the relative intensity of the peak corresponding to the DMPO–OH signal. Right images show representative signals of control (0 mM), 1 mM, and 10 mM EtPy treatment. Although 10 mM EtPy significantly reduced hydroxyl radicals, 1 mM of EtPy did not show significant effects when quantitatively evaluated using the spectra. Each bar represents mean ± SEM, n = 5. ** *p ≤* 0.01, compared with the 0 mM group.

As shown in Fig. 7A, 1 and 10 mm EtPy slightly scavenged peroxynitrite, reducing levels by approximately 10%. Fig. 7B shows data for the radical scavenging activity of EtPy as measured by EPR. The PR signals generated by the H_2_O_2_/UV radiation system showed typical •OH signals and were slightly blocked by 10 mM EtPy.

## 4 Discussion

EtPy has been identified as a possible antidote against APAP-induced hepatotoxicity in mouse models [18]. However, the precise mechanism of the *in vivo* hepatoprotective action of EtPy remains unclear. In this study, we confirmed that EtPy prevented serum transaminase elevation, hepatocellular centrilobular necrosis, and DNA fragmentation, but did not prevent nitrotyrosine adduct formation, induced by APAP in mice. The results support the previous report by Yang et al. [18], and suggest the preventive mode by which EtPy attenuates APAP hepatotoxicity, at least in part, through the inhibition of hepatocellular injury without oxidative stress.

Another objective of this study was to assess whether EtPy has a direct hepatocellular protective effect against cellular injury induced by NAPQI, a highly reactive toxic metabolite of APAP, in an *in vitro* hepatocellular experimental system. In this study, we found that EtPy prevented changes in cellular injury parameters, such as mitochondria dehydrogenase inactivity, and increases in annexin V and propidium iodide fluorescence intensities induced by NAPQI treatment in HepG2 cells. The results indicate that EtPy has protective potential against cellular injury induced by NAPQI in cultured hepatic cell models.

Numerous previous studies have demonstrated the potent anti-inflammatory potential of EtPy [19, 23]. EtPy can inhibit the release of cytokines from macrophages, such as TNF-α and IL-6 [19], and reactive oxygen species [29, 30], which are associated with the development of APAP-induced liver injury in mice [18]. Therefore, these anti-inflammatory properties of EtPy seem to play an important role in the attenuation of hepatitis observed in APAP-overdosed mice; that is “indirect” hepatocyte protection of EtPy exerted through inhibition of macrophages, cytokines, and reactive oxygen species.

Oxidative stress is an important factor in APAP hepatotoxicity, and mitochondrial oxidative stress seems to play a critical role in the development of hepatotoxicity [9]. Jaeschke et al. [31, 32] reported that nitrotyrosine formation is a marker of mitochondrial oxidative stress in APAP-induced liver injury. Nitric oxide reacts with superoxide in mitochondria and forms the reactive nitrogen peroxynitrite, which can then bind to the tyrosine residue of cellular proteins and develop into nitrotyrosine. Previously, we found that hydrophilic C60(OH)10 nanoparticles can attenuate APAP hepatotoxicity via a potent scavenger action against reactive oxygen and nitrogen, including peroxynitrite [26]. In this study, although extensive nitrotyrosine formation was observed in hepatocytes around central veins, EtPy did not inhibit nitrotyrosine formation in the mouse liver. Consistent with previous reports, we also found that EtPy showed a scavenger effect against peroxynitrite and hydroxyl radicals. Although the effects were statistically significant, higher concentrations were needed than the concentration required to exert a cytoprotective effect. From this, we consider that peroxynitrite and hydroxyl radicals may not be a main target molecule of EtPy in the protection against APAP hepatotoxicity. Further studies are needed on this point.

The results also indicate that inhibition of hepatocellular death induced by NAPQI may also be involved in antidote potential of EtPy against APAP hepatitis. The preventive effects of EtPy against NAPQI-induced cellular injury were comparable with the effects of NAC, an approved drug for APAP hepatitis, in this study. The results also suggest that EtPy is an attractive therapeutic candidate against APAP hepatotoxicity.

NAC seems to detoxify NAPQI toxicity through glutathione supplementation and an anti-oxidative action during APAP hepatotoxicity [33, 34, 35]. Wang et al. [22] demonstrated that EtPy has a preventive action on dopamine-induced cell death in PC12 cells, which may be because of the anti-apoptosis action of EtPy. Some *in vivo* studies suggest anti-apoptotic and/or necrotic effects of EtPy. In the current study, EtPy inhibited the increase in annexin V- and/or propidium iodide-stained cells in NAPQI-treated HepG2 cells. Annexin V stain is a representative marker for apoptosis- or necrosis-like dead cells. Therefore, EtPy may attenuate cellular injury induced by NAPQI through the inhibition of an apoptosis- or necrosis-like cell death pathway.

PyA, a parent compound of EtPy, and PEP, a precursor of PyA, have been demonstrated to show anti-inflammatory and anti-oxidative potential in previous studies [28, 36, 37, 38]. Although PEP did not show significant protection against NAPQI-induced cellular injury, PyA tended to attenuate the cytotoxicity induced by NAPQI in the current study. Therefore, the protective action of EtPy might be derived from the parent compound, PyA.

It is known that PyA rapidly forms parapyruvate, a potent inhibitor of the Krebs cycle, via an aldol-like condensation reaction in aqueous solution [23]. Therefore, the use of PyA *per se* as a therapeutic reagent is problematic; EtPy has been developed to circumvent this problem. Indeed, EtPy has been demonstrated to be a suitable derivative with the inherent advantages of PyA as an anti-inflammatory and anti-oxidative drug [21, 23]. Clinical trials on EtPy have been performed to evaluate the safety and therapeutic effects in high-risk cardiac surgery patients with systemic inflammation and in horses [39, 40]. Although no significant therapeutic benefit has been identified in clinical trials to date, the possibility of the clinical use of EtPy cannot be denied because of its safety observed in the trials. Further studies are necessary to evaluate the clinical benefits in other diseases. From previous and current findings, we suggest that EtPy should be investigated for its potential benefits as a new drug in patients with APAP overdose.

In this study, while we observed the attenuating effects of EtPy in mouse and cellular models of APAP-induced liver injury, the study does have some limitations. First, the protective mechanism of EtPy against APAP hepatotoxicity was not fully clarified in this study. We first thought that inhibition of oxidative stress, particularly with regard to nitrotyrosine formation, was a central part of the protective mechanism of EtPy. However, treatment with EtPy did not inhibit nitrotyrosine formation in the mouse liver treated with APAP. Therefore, we now consider that other factors in APAP hepatotoxicity, such as mitophagy [41, 42], endoplasmic reticulum stress [13, 43], DNA fragmentation by endonuclease G, and apoptosis-inducing factor [31, 44, 45], may be critical targets of EtPy. Further studies are needed to clarify the precise mechanism. Second, we used mitochondrial dehydrogenase activity as a measure of cellular dysfunction. Although mitochondrial dehydrogenase activity measured using WST or MTT assays has been used as a parameter in viable cells, it does not always reflect cell viability. Studies have indicated that cellular mitochondrial dehydrogenase activity was inhibited without cell death under certain experimental conditions [46, 47, 48]. Indeed, we also observed inconsistencies in the results between mitochondrial dehydrogenase activity and the annexin V/PI assay in this study. Therefore, the decrease in mitochondrial dehydrogenase activity observed in NAPQI-treated cells may indicate other phenomena, such as mitochondrial dysfunction, rather than only cell death. Third, to evaluate the direct hepatoprotective effect of EtPy against NAPQI-induced toxicity, we used immortalized human hepatoma HepG2 cells for the hepatocyte model. Although HepG2 cells are used as a hepatocellular model, there seems to be some weakness when used in an APAP hepatotoxicity model [49]. HepG2 cells show lower expression of CYP2E1 and do not react well to APAP treatment. Antidote NAC cannot protect HepG2 cell injury induced by APAP [50]. Therefore, the mode of cell death in HepG2 cells induced by APAP was quite different when compared with that of primary cultured hepatocytes [49]. In the current study, we used an active metabolite NAPQI instead of APAP to induce cytotoxicity in HepG2 cells, and observed preventive effects with NAC as well as EtPy. The cytoprotective action of EtPy and NAC was also confirmed in another cell line (RLC cells). Even though the experimental system using NAPQI does have some limitations, the results suggest that EtPy has cytoprotective potential against NAPQI in cells. Further studies are needed to determine the direct hepatocyte protective effect of EtPy and the mode of action using other models, such as human primary hepatocytes or pluripotent stem cell derived hepatocytes [51].

## 5 Conclusion

We confirmed that EtPy showed hepatoprotective action against APAP hepatitis in mice. Although hepatic DNA fragmentation was inhibited with EtPy treatment in mice, nitrotyrosine formation was not prevented. EtPy also significantly attenuated cellular injury induced by NAPQI, an active toxic metabolite of APAP, in hepatic cell lines. The results suggest that the protective potential of EtPy against APAP hepatotoxicity observed in animal models is exerted, at least in part, through direct hepatocellular protection against NAPQI.

## Declarations

### Author contributions statement

Minako Nagatome, Yuki Kondo: Conceived and designed the experiments; Performed the experiments; Analyzed and interpreted the data.

Mitsuru Irikura, Tetsumi Irie: Conceived and designed the experiments.

Yoichi Ishitsuka: Conceived and designed the experiments; Performed the experiments; Analyzed and interpreted the data; Wrote the paper.

### Competing interest statement

The authors declare that they have no competing interests.

### Funding statement

This work was supported by the Japan Society for the Promotion of Science (Grant-in-Aid for Scientific Research, JSPS KAKENHI, Grant No. 21790524, 23790603, and 26460629 to Y Ishitsuka).

### Additional information

No additional information is available for this paper.
